# Validity and predictors of BMI derived from self-reported height and weight among 11- to 17-year-old German adolescents from the KiGGS study

**DOI:** 10.1186/1756-0500-4-414

**Published:** 2011-10-17

**Authors:** Anna-Kristin Brettschneider, Angelika Schaffrath Rosario, Ute Ellert

**Affiliations:** 1 Department of Epidemiology and Health Reporting, Robert Koch Institute, General-Pape-Str. 62-66, 12101 Berlin, Germany

## Abstract

**Background:**

For practical and financial reasons, self-reported instead of measured height and weight are often used. The aim of this study is to evaluate the validity of self-reports and to identify potential predictors of the validity of body mass index (BMI) derived from self-reported height and weight.

**Findings:**

Self-reported and measured data were collected from a sub-sample (3,468 adolescents aged 11-17) from the German Health Interview and Examination Survey for Children and Adolescents (KiGGS). BMI was calculated from both reported and measured values, and these were compared in descriptive analyses. Linear regression models with BMI difference (self-reported minus measured) and logistic regression models with weight status misclassifications as dependent variables were calculated.

Height was overestimated by 14- to 17-year-olds. Overall, boys and girls under-reported their weight. On average, BMI values calculated from self-reports were lower than those calculated from measured values. This underestimation of BMI led to a bias in the prevalence rates of under- and overweight which was stronger in girls than in boys. Based on self-reports, the prevalence was 9.7% for underweight and 15.1% for overweight. However, according to measured data the corresponding rates were 7.5% and 17.7%, respectively. Linear regression for BMI difference showed significant differences according to measured weight status: BMI was overestimated by underweight adolescents and underestimated by overweight adolescents. When weight status was excluded from the model, body perception was statistically significant: Adolescents who regarded themselves as 'too fat' underestimated their BMI to a greater extent. Symptoms of a potential eating disorder, sexual maturation, socio-economic status (SES), school type, migration background and parental overweight showed no association with the BMI difference, but parental overweight was a consistent predictor of the misclassification of weight status defined by self-reports.

**Conclusions:**

The present findings demonstrate that the observed discrepancy between self-reported and measured height and weight leads to inaccurate estimates of the prevalence of under- and overweight when based on self-reports. The collection of body perception data and parents' height and weight is therefore recommended in addition to self-reports. Use of a correction formula seems reasonable in order to correct for differences between self-reported and measured data.

## Background

The prevalence of overweight and obesity has increased worldwide in recent years. Especially for children and adolescents, this issue has emerged as a major public health concern [1]. In Germany, the proportion of overweight and obese children and adolescents aged between 3 and 17 has risen by about 50% compared to the early 1990s. The percentage of overweight (including obese) adolescents aged 11-17 has almost doubled, and the prevalence of obesity has nearly tripled [2]. Overweight and obesity can act as predictor of various diseases. In the short term it is associated with several cardiovascular risk factors and mental health problems, e.g. low self-esteem [3,4]. In terms of long-term consequences, obesity in childhood is a predictor of adult obesity. Increased mortality and morbidity can be consequences of overweight and obesity in adulthood [5]. Continuous monitoring of weight status in the population therefore needs to be established. For practical and financial reasons, large-scale studies frequently use self-reported height and weight assessed via self-administered questionnaires or telephone interviews.

A number of previous studies have examined the validity of self-reported height and weight among children and adolescents. Results for height were mixed; most studies reported a systematic overestimation of height [6-20], while some found underestimation or no difference [21-25]. Weight, on the other hand, was consistently under-reported [6-28]. This resulted in a lower body mass index (BMI) and led to a bias in the prevalence rates of under- and overweight compared to prevalence rates based on measured values [6-16,18-20,22,24,26-28]. The results of previous studies vary with respect to gender differences. Mostly, height was more often over-reported and weight more often under-reported by girls than by boys [6,9,12,16,23,28]. Some previous studies reported age-related differences in self-reports of height and weight [8,15,16,21].

Underweight children and adolescents over-reported their weight [7], while normal weight and overweight/obese children tended to underestimate their weight. Differences between measured and self-reported weight - and consequently BMI - were significantly greater for overweight/obese children and adolescents compared to subjects of normal weight [7,9,11,12,15,16,22,24,25,27]. Some studies have examined whether body perception has an influence on self-reports. Boys and girls who felt 'too fat' or wished to be leaner under-reported their weight to a greater extent than those who were satisfied with their body size [9,22]. Several studies have also considered the effects of ethnicity and socio-economic status (SES) or school type [6,8,9,17,20,22,24,28], but few have analysed other potential predictors of the validity of self-reports [20].

The purpose of the present study was, first, to evaluate the validity of BMI derived from self-reported height and weight in a sample of 11- to 17-year-old adolescents, and, second, to identify potential predictors of the validity of BMI calculated from self-reported height and weight.

## Findings

### Methods

#### Study population

The data used in this study is based on a subset of the German Health Interview and Examination Survey for Children and Adolescents (KiGGS). KiGGS is a cross-sectional study which was conducted from May 2003 to May 2006 in order to collect comprehensive data on the health status of children and adolescents aged 0-17. A total of 17,641 boys and girls from 167 study locations representative of Germany were surveyed (response rate 66.6%). Participants over the age of 14 and all parents/caregivers gave their written informed consent prior to the interview and examination. The survey was approved by the Federal Office for Data Protection and by the ethics committee of Charité Universitätsmedizin Berlin.

Among other methods, self-administered questionnaires filled in by parents, parallel questionnaires for adolescents aged 11 and older, and physical examinations were used in the survey [29]. Self-reports of height and weight were collected face-to-face and only in the second half of the survey (starting in November 2004). Participants with implausible or missing values for measured or self-reported height and weight were excluded from the analysis. Measured height data was missing for 3 and measured weight data for 23 adolescents. Self-reported height (weight) was missing for 181 (169) adolescents; in 2 adolescents the self-reports were not plausible.

#### Anthropometric measurements and self-reports

Anthropometric measurements were taken by trained staff using standardized methods. Body height was measured without shoes to an accuracy of 0.1 cm using a portable Harpenden stadiometer (Holtain Ltd., UK). Body weight was measured to the nearest 0.1 kg, wearing underwear, using a calibrated electronic scale (SECA Ltd., Germany).

Prior to the standardized measurement, adolescents were asked face-to-face to report their height (without shoes) and weight (without clothes) to an accuracy of 1 cm or 1 kg, respectively.

Body mass index (BMI) in kg/m^2 ^was calculated both from self-reported and from measured data. Weight status was determined using age- and gender-specific cut-offs for underweight (<10^th ^percentile), normal weight (≥10^th ^percentile to ≤90^th ^percentile) and overweight (>90^th ^percentile) based on the national German reference [30]. Throughout this paper, the category 'overweight' includes obese adolescents.

#### Potential predictors of the quality of self-reported height, weight and BMI

Each adolescent's body perception was examined by asking the following question in the self-administered questionnaire: 'Do you think you are ...' 'much too thin', 'a bit too thin', 'exactly the right weight', 'a bit too fat', or 'much too fat'? Responses were classified into the following categories: (1) 'too thin', (2) 'right weight', and (3) 'too fat'.

The SCOFF 5-question screening instrument, which addresses the core features of anorexia nervosa and bulimia nervosa, was used to detect symptoms of eating disorders [31]. Two positively answered questions were considered suspicious of an eating disorder.

Information on sexual maturation status was obtained by the self-assessment of pubic hair status according to standardized drawings (Tanner stages) [32]. They were classified into the following categories: (1) infantile (Tanner stage 1), (2) early puberty (Tanner stage 2-3), and (3) late or post puberty (Tanner stage 4-6).

Data taken from the parental questionnaire on the parents' income, occupational status, and educational and occupational qualification was used to quantify the SES of the adolescents. Each of the three components was rated using a point system (1-7 points). The sum was calculated and categorized into the following groups: (1) low SES (3-8 points), (2) medium SES (9-14 points), and (3) high SES (15-21 points) [33]. Participants were referred to as immigrants if they themselves had immigrated and had at least one parent who was not born in Germany or was of non-German nationality, or if both parents had immigrated or were of non-German nationality [34]. Self-reported height and weight of mothers and fathers were used to calculate parental BMI, which was classified into overweight (yes/no) according to the WHO cut-off point of ≥ 25 kg/m^2 ^[1]. They were allocated to the following categories: (1) both parents overweight, (2) one parent overweight (including single parents who are overweight), and (3) no parent overweight.

#### Statistical analysis

SPSS 14 for Windows (Chicago, Illinois) was used for data management and statistical calculations. The sample of 3,468 adolescents was tabulated according to age groups (11-13 and 14-17) and gender. The differences between self-reported and measured values for height, weight and BMI were calculated and tested for difference from zero using the paired samples t-test. The strength of the relationship between self-reported and measured values was described by Cohen's d as ≤0.2, >0.2 to <0.8, and ≥0.8 indicating a small, medium, and large effect, respectively [35]. Differences between boys and girls or between age groups were also tested using Student's t-test. Bland-Altman plots were used to visualize the agreement of self-reported and measured data [36]. Sensitivity and specificity for underweight, normal weight and overweight were assessed and expressed as percentages. Confidence intervals (CI) for sensitivity and specificity were calculated via the normal approximation to the binomial distribution. The difference in the prevalence of self-reported vs. measured overweight was assessed using the McNemar test for paired data [37].

In linear regression models adjusted for age (in years, as a categorical variable), the association of potential predictors of the validity of self-reports with the difference between self-reported and measured BMI was examined, separately for boys and girls. All variables which showed significance in univariate linear regression models were included in a multivariate model. The multivariate model was run in two versions: Model 1a including weight status based on measured BMI as an independent variable, and Model 1b without this variable. Model 1b simulates the situation in a study where only self-reports are available. In this situation, it is important to know how self-reports differ from measured values and which variables predict these differences, independent of the actual weight status.

Logistic regression models were estimated in order to identify potential predictors for a misclassification of weight status. Misclassification was defined as discordance between weight status determined by measured and self-reported data. Four different models based on different analysis populations were built:

Model 2a: Includes all overweight adolescents, with the target variable 'overweight misclassified by self-reports as normal weight or underweight'. This is equal to 1-sensitivity for overweight.

Model 2b: Includes all normal weight adolescents, with the target variable 'normal weight misclassified by self-reports as overweight'.

Model 2c: Includes all normal weight adolescents, with the target variable 'normal weight misclassified by self-reports as underweight'.

Model 2d: Includes all underweight adolescents, with the target variable 'underweight misclassified by self-reports as normal weight or overweight'. This is equal to 1-sensitivity for underweight.

A secondary analysis with type of school (low, moderate, high) as potential predictor (instead of SES) in linear and logistic regression models was conducted.

All variables that showed significance in univariate logistic regression models were included in a multivariate model. Because of the small absolute number of misclassified cases, the logistic regression models were not conducted separately for gender, but adjusted for gender and age. For the same reason, age and SES were entered as continuous variables.

Survey weights [29] were not used in the analysis, since only data from the second half of the survey were included.

## Results

**Figure 1:**
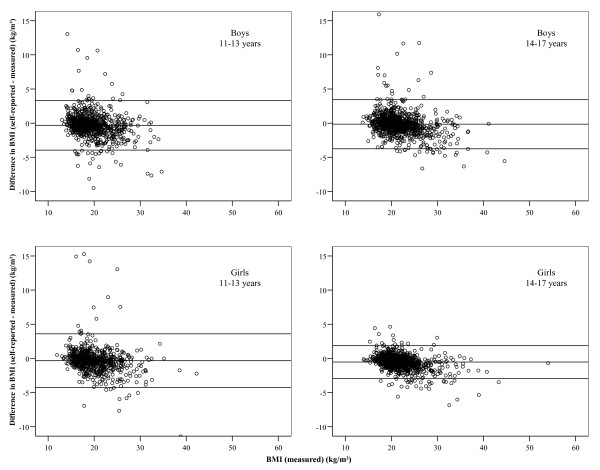
**Bland-Altman plots of differences between self-reported and measured BMI (3 extremes not displayed)**.

The subsample analysed comprised 3,468 adolescents (1,792 boys and 1,676 girls) aged 11-17. Characteristics of the study population are shown in Table 1. Girls were almost twice as likely to show symptoms of an eating disorder, to be less satisfied with their body shape than boys (55% feeling 'too fat' compared to 36% in boys), and to be more advanced in their sexual maturation, while measured prevalence rates for overweight were identical in boys and girls. Differences between self-reported and measured height and weight varied by gender and by age group as shown in Table 2. Height differences were not statistically significantly different from zero in 11- to 13-year-olds, but height was overestimated by 14- to 17-year-old adolescents. In 14- to 17-year-olds, overestimation of height was higher in girls than in boys (*p *< 0.001). Overall, boys and girls under-reported weight, the underestimation being stronger among 11- to 13-year-olds (p = 0.001 and *p *< 0.01, respectively). In 14- to 17-year-olds, girls under-reported their weight to a larger extent than boys (*p *< 0.05). The average difference between self-reported and measured height and weight corresponds to 0.7% of the mean value for height and to 1.1% of the mean value for weight. On average, BMI values calculated from self-reported height and weight were lower than BMI values calculated from measured data. In 11- to 13-year-olds, there was no gender difference, while 14- to 17-year-old girls underestimated their BMI more strongly than boys (*p *< 0.001) and more strongly than 11- to 13-year-old girls (*p *= 0.01), but they had the lowest standard deviation in the differences and showed fewer gross differences. Cohen's d indicated a small effect (<0.20) for all types of differences. The Bland-Altman plots, shown in Figure 1, suggest a general tendency for a stronger underestimation with increasing BMI.

**Table 1:** Description of study population

	Boys (n = 1 792)		Girls (n = 1 676)		*p*^*1*^	Total (n = 3 468)	
	N	%	N	%		N	%
**Age**					0.435		
11-13	802	**44.8**	728	**43.4**		1 530	**44.1**
14-17	990	**55.2**	948	**56.6**		1 938	**55.9**
**Weight status (measured)**					0.381		
Underweight	145	**8.1**	115	**6.9**		260	**7.5**
Normal weight	1 329	**74.2**	1 264	**75.4**		2 593	**74.8**
Overweight	318	**17.7**	297	**17.7**		615	**17.7**
**Weight status (self-reported)**					0.339		
Underweight	179	**10.0**	159	**9.5**		338	**9.7**
Normal weight	1 328	**74.1**	1 277	**76.2**		2 605	**75.1**
Overweight	285	**15.9**	240	**14.3**		525	**15.1**
**Body perception**					0.000		
Too thin	339	**19.2**	149	**9.0**		488	**14.2**
Right weight	797	**45.0**	605	**36.4**		1 402	**40.9**
Too fat	634	**35.8**	907	**54.6**		1 541	**44.9**
Missing	22		15			37	
**Symptoms of an eating disorder**					0.000		
Conspicuous	284	**16.1**	480	**29.0**		764	**22.4**
Inconspicuous	1 476	**83.9**	1 176	**71.0**		2 652	**77.6**
Missing	32		20			52	
**Sexual maturation**					0.000		
Tanner 1	106	**6.0**	81	**4.9**		187	**5.5**
Tanner 2-3	532	**30.3**	244	**14.7**		776	**22.7**
Tanner 4-6	1 120	**64.0**	1 335	**80.0**		2 455	**71.8**
Missing	34		16			50	
**Socio-economic status**					0.934		
Low	473	**27.3**	440	**26.8**		913	**27.1**
Moderate	844	**48.8**	801	**48.8**		1 645	**48.8**
High	414	**23.9**	399	**24.3**		813	**24.1**
Missing	61		36			97	
**Migration background**					0.206		
Migrant	281	**15.7**	237	**14.1**		518	**14.9**
Non-migrant	1 511	**84.3**	1 438	**85.9**		2 949	**85.1**
Missing	0		1			1	
**Parental overweight**					0.495		
Both	422	**24.4**	374	**22.9**		796	**23.6**
One	790	**45.6**	770	**47.1**		1 560	**46.3**
None	520	**30.0**	492	**30.1**		1 012	**30.0**
Missing	60		40			100	

^*1 *^*Pearson's Chi-square Test*

**Table 2:** Mean, standard deviation (SD), *p*-value, Cohen's *d *and 95% CI of measured and self-reported data

Boys	11-13 years (n = 802)					14-17 years (n = 990)				
	Mean	SD	*p*^*1*^	*d*^*2*^	95% CI	Mean	SD	*p*^*1*^	*d*^*2*^	95% CI
**Measured data**										
Height (cm)	**156.2**	10.01			155.53-156.92	**175.0**	8.54			174.43-175.50
Weight (kg)	**48.4**	12.65			47.52-49.28	**67.1**	14.43			66.16-67.96
BMI (kg/m^2^)	**19.6**	3.64			19.35-19.86	**21.8**	3.95			21.56-22.05
**Self-reported data**										
Height (cm)	**156.2**	10.78			155.44-156.93	**175.3**	9.54			174.66-175.85
Weight (kg)	**47.5**	12.17			46.64-48.32	**66.8**	14.19			65.89-67.66
BMI (kg/m^2^)	**19.3**	3.64			19.04-19.54	**21.7**	3.85			21.41-21.89
**Difference between self-reported and measured data**										
Height (cm)	**-0.04**	4.82	0.825	0.00	-0.37-0.30	**0.29**	4.07	0.025	0.03	0.04-0.54
Weight (kg)	**-0.92**	3.41	0.000	0.07	-1.15-(-0.68)	**-0.28**	4.37	0.041	0.02	-0.56-(-0.01)
BMI (kg/m^2^)	**-0.32**	1.85	0.000	0.09	-0.45-(-0.19)	**-0.15**	1.84	0.008	0.04	-0.27-(-0.04)

**Girls**	**11-13 years **(n = 728)					**14-17 years **(n = 948)				
	**Mean**	SD	*p*^*1*^	*d*^*2*^	95% CI	**Mean**	SD	*p*^*1*^	*d*^*2*^	95% CI
**Measured data**										
Height (cm)	**156.6**	8.44			155.99-157.21	**164.8**	6.38			164.37-165.19
Weight (kg)	**49.7**	12.49			48.82-50.64	**59.7**	11.57			58.91-60.38
BMI (kg/m^2^)	**20.1**	3.92			19.81-20.38	**21.9**	3.91			21.68-22.18
**Self-reported data**										
Height (cm)	**156.3**	10.08			155.53-157.00	**166.0**	6.84			165.53-166.40
Weight (kg)	**48.7**	12.58			47.74-49.57	**59.0**	11.20			58.29-59.72
BMI (kg/m^2^)	**19.8**	3.98			19.48-20.06	**21.4**	3.68			21.16-21.62
**Difference between self-reported and measured data**										
Height (cm)	**-0.33**	4.80	0.060	0.04	-0.68-0.01	**1.18**	2.70	0.000	0.18	1.01-1.35
Weight (kg)	**-1.07**	4.08	0.000	0.09	-1.37-(-0.77)	**-0.64**	2.45	0.000	0.06	-0.80-(-0.48)
BMI (kg/m^2^)	**-0.33**	2.00	0.000	0.08	-0.48-(-0.18)	**-0.54**	1.23	0.000	0.14	-0.62-(-0.47)

^1 ^paired samples t-test for difference from zero

^2 ^Cohen's d value calculated as effect size

Based on self-reported data, prevalence estimates were 9.7% (95% CI 8.7-10.7) for underweight, 75.1% (95% CI 73.7-76.5) for normal weight, and 15.1% (95% CI 13.9-16.3) for overweight. However, according to measured data the corresponding rates were 7.5% (95% CI 6.6-8.4) for underweight, 74.8% (95% CI 73.4-76.2) for normal weight, and 17.7% (95% CI 16.4-19.0) for overweight. The difference in prevalence rates for overweight was higher for girls (14.3% self-reported vs. 17.7% measured) than for boys (15.9% vs. 17.7%), but was statistically significant in both boys (McNemar *p *= 0.003) and girls (McNemar *p *< 0.001). Sensitivity for overweight was 75.8% for boys and 73.7% for girls; specificity was 97.0% for boys and 98.5% for girls (see Table 3 for CI and the values for under- and normal weight).

**Table 3:** Sensitivity and specificity for weight status by gender

		Sensitivity								Specificity						
		Boys				Girls				Boys				Girls		
		%		95% CI		%		95% CI		%		95% CI		%		95% CI
**Weight status**																
Underweight		**66.9**		59.2-74.6		**76.5**		68.8-84.2		**95.0**		91.5-98.5		**95.5**		91.7-99.3
Normal weight		**90.7**		89.1-92.3		**92.7**		91.3-94.1		**73.7**		71.3-76.1		**74.5**		72.1-76.9
Overweight		**75.8**		71.1-80.5		**73.7**		68.7-78.7		**97.0**		95.1-98.9		**98.5**		97.1-99.9

Univariate linear regression models (adjusted for age) confirmed that BMI was overall under-reported to a lesser extent by boys than by girls (*p *< 0.001), with 14- to 17-year-old girls showing the largest bias (data not shown). Sexual maturation, SES, type of school, and migration background showed no significant association with the difference between self-reported and measured BMI in univariate models (data not shown). Table 4 shows the results of the multivariate linear regression for BMI difference with the potential predictors age, weight status, body perception, symptoms of an eating disorder, and parental overweight. For boys, age was a significant predictor of the difference between self-reported and measured BMI (*p *< 0.05). The multivariate model including weight status as independent variable (Model 1a) showed that underweight boys over-reported their BMI compared to normal weight boys, whereas BMI was under-reported by overweight boys. No significant association between BMI difference and body perception could be seen in this model. However, in the multivariate model not including weight status (Model 1b), boys with the body perception 'too fat' under-reported their BMI compared to boys who considered themselves 'the right size'. Boys who considered themselves 'too thin' over-reported their BMI, but this estimate was not statistically significant (*p *= 0.10). For girls, age had a statistically significant predictive effect on the difference between self-reported and measured BMI (*p *< 0.05) in Model 1a, but only borderline significance in Model 1b (*p *= 0.07). In Model 1a, underweight girls over-reported their BMI compared to normal weight girls, whereas BMI was under-reported by overweight girls. Even controlled for weight status, girls perceiving their body as 'too fat' under-reported their BMI by -0.2 kg/m^2 ^compared to normal weight girls, with borderline statistical significance (*p *= 0.06). In Model 1b, results were similar to those for boys: Girls who felt 'too fat' significantly under-reported their BMI compared to normal weight girls, while the over-reporting in girls perceiving their body as 'too thin' was not statistically significant (*p *= 0.09). Symptoms of an eating disorder and parental overweight were not statistically significantly associated with the difference between measured and reported BMI in any model (Table 4). The R-squared values in linear regression models were low; the models explained only 4-7% of the variability in the absolute difference between self-reported and measured BMI. In Model 1a the values were higher than in Model 1b.

**Table 4:** Summary of multivariate linear regression models for the difference between self-reported and measured BMI

	Boys (n = 1 792)				Girls (n = 1 676)			
	Model 1a		Model 1b		Model 1a		Model 1b	
	B	*p*	B	*p*	B	*p*	B	*p*
**Intercept**	-0.39		-0.34		-0.25		-0.27	
**Age **(in years)								
11	0.00	ref.	0.00	ref.	0.00	ref.	0.00	ref.
12	0.18	0.250	0.23	0.140	0.31	0.025	0.32	0.022
13	0.36	0.021	0.39	0.015	0.06	0.687	0.06	0.669
14	0.49	0.002	0.49	0.002	-0.08	0.572	-0.03	0.838
15	0.34	0.031	0.31	0.058	-0.03	0.839	0.05	0.741
16	0.45	0.006	0.45	0.006	-0.20	0.160	-0.14	0.338
17	0.22	0.194	0.21	0.225	-0.07	0.647	0.02	0.884
**Weight status (measured)**								
Underweight	0.64	0.000	not included in the model		0.37	0.028	not included in the model	
Overweight	-0.87	0.000			-0.67	0.000		
Normal weight	0.00	ref.			0.00	ref.		
**Body perception**								
Too thin	0.03	0.825	0.20	0.101	0.10	0.531	0.24	0.085
Too fat	-0.11	0.360	-0.46	0.000	-0.17	0.057	-0.38	0.000
Right weight	0.00	ref.	0.00	ref.	0.00	ref.	0.00	ref.
**Symptoms of an eating disorder**								
Conspicous	-0.07	0.572	-0.15	0.231	-0.12	0.171	-0.16	0.072
Inconspicous	0.00	ref.	0.00	ref.	0.00	ref.	0.00	ref.
**Parental overweight**								
Both	-0.08	0.487	-0.21	0.084	not significant in the univariate model		not significant in the univariate model	
One	0.06	0.581	0.01	0.943				
None	0.00	ref.	0.00	ref.				
**R^2^**	0.068		0.037		0.062		0.037	

Model 1a: Multivariate model with measured weight status

Model 1b: Multivariate model without measured weight status

ref.= reference

Table 5 shows the results of the logistic regression models (Model 2a-2c). Model 2a showed that overweight adolescents who felt 'too fat' had 72% (95% CI 0.46-0.86) lower odds to be classified as normal weight or underweight by their self-reports than overweight adolescents who felt they had the 'right weight'. The higher the social status, the higher the odds that overweight was misclassified as normal weight or underweight (OR = 1.06 per index point). Overweight adolescents with two overweight parents had 53% (95% CI 0.18-0.72) lower odds to be classified as normal weight or underweight by their self-reports than overweight participants with normal weight parents. Age, gender, symptoms of an eating disorder, maturation status, type of school and migration background had no statistically significant association with the misclassification of overweight adolescents. Model 2b showed that normal weight boys have 2.68fold (95% CI 1.50-4.80) higher odds to be misclassified as overweight than normal weight girls. Normal weight adolescents who described themselves as 'too fat' had 2.2fold (95% CI 1.25-3.87) higher odds to be classified as overweight than those who considered themselves to be of 'the right weight'. Normal weight adolescents with a migration background had 2.36fold (95% CI 1.28-4.34) higher odds to misclassify themselves as overweight. The odds that normal weight adolescents were classified as overweight by self-reports were also higher if they had overweight parents, especially when both parents were overweight. The secondary analysis with type of school instead of SES showed that normal weight adolescents who visited a low level school had 2.8fold (95% CI 1.34-5.68) higher odds to be classified as overweight than adolescents who visited a high level school. Age, symptoms of an eating disorder, maturation status and SES had no statistically significant odds ratios in this model. Model 2c included normal weight adolescents where the target variable was the misclassification of normal weight as underweight. Normal weight boys had 31% (95% CI 0.01-0.52) lower odds to be classified as underweight by their self-reports than girls. Normal weight boys and girls who thought they were 'too thin' had 2.97fold (95% CI 1.99-4.43) increased odds to be misclassified as underweight compared to those who felt 'the right weight', whereas a body perception of 'too fat' decreased these odds by 72% (95% CI 0.52-0.84). The less sexual maturation was advanced, the higher were the odds to be misclassified as underweight. Age, symptoms of an eating disorder, SES, type of school and migration background showed no significant association with the misclassification of normal weight as underweight.

**Table 5:** Summary of multivariate logistic regression models

	Model 2a (n = 615)			Model 2b (n = 2 593)			Model 2c (n = 2 593)		
	Odds ratio	95% CI	*p*	Odds ratio	95% CI	*p*	Odds ratio	95% CI	*p*
**Age **(per year)	1.04	0.95-1.15	0.387	0.92	0.81-1.06	0.249	0.97	0.86-1.09	0.588
**Gender**									
Boys	0.78	0.53-1.15	0.210	2.68	1.50-4.80	0.001	0.69	0.48-0.99	0.045
Girls	1.00	reference	reference	1.00	reference	reference	1.00	reference	reference
**Body perception**									
Too thin	-	-	-	0.63	0.21-1.85	0.399	2.97	1.99-4.43	0.000
Too fat	0.28	0.14-0.54	0.000	2.20	1.25-3.87	0.006	0.28	0.16-0.48	0.000
Right weight	1.00	reference	reference	1.00	reference	reference	1.00	reference	reference
**Symptoms of an eating disorder**									
Conspicuous	0.78	0.52-1.18	0.241	not significant in the univariate model			not significant in the univariate model		
Inconspicuous	1.00	reference	reference						
**Sexual maturation**									
Tanner 1							2.48	1.20-5.13	0.014
Tanner 2-3	not significant in the univariate model			not significant in the univariate model			2.40	1.46-3.94	0.001
Tanner 4-6							1.00	reference	reference
**SES**									
Per index point	1.06	1.01-1.11	0.021	not significant in the univariate model			not significant in the univariate model		
**Migrant background**									
Migrant	not significant in the univariate model			2.36	1.28-4.34	0.006	not significant in the univariate model		
Non-migrant				1.00	reference	reference			
**Parental overweight**									
Both	0.47	0.28-0.82	0.007	4.16	1.75-9.88	0.001	0.49	0.28-0.86	0.013
One	0.64	0.38-1.06	0.085	2.71	1.18-6.24	0.019	0.84	0.58-1.21	0.343
None	1.00	reference	reference	1.00	reference	reference	1.00	reference	reference
**Pseudo R^2^**	0.077			0.083			0.122		

Model 2a includes all overweight individuals, with the target variable 'overweight misclassified by self-reports as normal weight or underweight' (n = 77 individuals misclassified).

Model 2b includes all normal weight individuals, with the target variable 'normal weight misclassified by self-reports as overweight' (n = 42 individuals misclassified).

Model 2c includes all normal weight individuals, with the target variable 'normal weight misclassified by self-reports as underweight' (n = 81 individuals misclassified).

No multivariate model was generated for underweight adolescents and the target variable 'misclassification of underweight as normal or overweight' (Model 2d), because no significant associations on the univariate level could be found.

## Discussion

The aim of this study was to evaluate the validity of BMI derived from self-reported height and weight in adolescents aged 11-17, and to identify potential predictors of the validity of BMI calculated from self-reported height and weight. The study demonstrated that the observed discrepancy between self-reported and measured height and weight led to inaccurate estimates of the prevalence of under- and overweight in Germany, if the estimates are based on self-reports. Although the bias in mean BMI differences was small, self-reports resulted in a considerable underestimation of BMI and thus a lower prevalence of overweight and a higher prevalence of underweight, especially in girls. The identified main predictors of the validity of the BMI self-reports in adolescents were gender, age, weight status, and body perception (the latter only in the absence of information about the actual weight status). In models where misclassification probability was a target variable, other potential predictors also had a predictive effect; the most consistent results were for parental overweight.

The results for sensitivity and specificity cannot be directly compared with other studies, since they were based on the national German cut-off points for defining under- and overweight, but like previous studies we found low values for sensitivity and higher values for specificity [6,9,17,26].

The descriptive analysis showed that differences between self-reported and measured values were on average larger in girls than in boys, as found by others [6,9,12,16,23,28]. One reason for this stronger misreporting in girls might be seen in the social desirability and social norms for thinness, which place a burden on girls in particular [6,11,23]. This desire was not just shown in their under-reporting of body weight, it was also apparent in their over-reporting of height. Girls might even know how BMI is calculated and that over-reporting height leads to a lower BMI, which could explain the stronger overestimation of height in girls aged 14-17 compared to boys. An alternative explanation might be that boys in this age group continue to grow at a faster pace than girls [38] and thus tend to report outdated height values. The significant BMI differences between self-reported and measured values, combined with the low standard deviation, suggest a systematic under-reporting of BMI by girls aged 14-17. Age-related differences were also seen in our study, as reported by others. Height was overestimated by 14- to 17-year-olds, as in the majority of preceding studies [6-17,21-28], whereas the under-reporting of height seen by Himes et al. (2001) and Jansen et al. (2006) was not confirmed in our study [21,22]. Weight was under-reported to a larger extent by adolescents aged 11-13 compared to 14- to 17-year-old boys and girls. One explanation might be the growth spurt, especially the higher weight gain in 11- to 13-year-olds [38], which may occur too rapidly for them to update their measurements.

Linear regression models showed that BMI was under-reported to the largest extent by overweight adolescents, as found by others [7,9,11,12,15,16,22,24,25,27].

This may also be associated with the desire to be leaner. The few studies that have considered self-reports by underweight adolescents confirmed the result of this study, i.e. that underweight adolescents tend to over-report their BMI [10,14]. When measured weight status was not included in the linear regression model, body perception was a major predictor of the quality of self-reports. Boys and girls who regarded themselves as 'too fat' under-reported their BMI significantly, as reported in previous studies [9,22]. But in the logistic regression model restricted to overweight adolescents, those who felt 'too fat' had lower odds to be misclassified as normal weight by their self-reports. Similarly, normal weight adolescents who described themselves as 'too fat' had lower odds to be misclassified as underweight, but higher odds to be misclassified as overweight by their self-reported height and weight. Thus, it seems that body perception can be a source of some information on the deviation of self-reports from actual weight status; it can also be used to correct self-reports to better approximate the actual weight status in situations where self-reports are of relevance, i.e. when no measured BMI values are available. Parental overweight also had a predictive effect on the probability of misclassification in some of the models. This may be related to the fact that overweight parents have heavier children [39], and if these children are near the border between normal weight and overweight, a misclassification is more likely, even if the BMI difference between the self-reported and measured values is small. The same mechanism can explain why a migration background, a lower SES or school type, or a more advanced stage of sexual maturation were associated with the misclassification probabilities in some of the models, as these groups had a higher risk of overweight [39-41]. The results for these variables were not as consistent as for parental overweight, however. Results for SES and migration background are difficult to compare with other studies, because most other studies did not use multivariate models.

The descriptive finding that girls tended to underestimate their BMI more strongly than boys was not reflected by higher regression coefficients in the multivariate linear regression models. It rather seems to be due to the higher proportion of girls who considered themselves to be 'too fat'.

This study has strengths and limitations. The first strength is that the self-reported and measured data were collected at the same time. A second advantage is the large sample size and the wide age range covered (11-17 years). A third strength is the high number of covariables that were examined in this study and included in multivariate models, e.g. the collection of the adolescents' body perception. An important limitation is the fact that the self-reported values were collected face-to-face, so that gross over- or underestimation was more difficult than in a written questionnaire. Furthermore, many adolescents in this study may have been aware that height and weight would be measured following the self-reports, because a description of the study procedures had been available to the participants on the internet beforehand. Another limitation is that we do not know whether the participants had measured their height and weight recently prior to the survey, so it is not known which ages the self-reported measurements in fact correspond to. This point is of particular importance in adolescents because of the changes in height and weight during pubertal development. Furthermore, the difference in accuracy between self-reports (to the nearest cm) and measurements (to the nearest mm) may in principle be a limitation. But a comparison of rounded with unrounded measured data lead to almost identical results.

## Conclusions

The use of self-reported height and weight leads to a bias in the prevalence rates for over- and underweight which is stronger in girls than in boys. Gender and age exert an influence on the patterns of accuracy of the self-reported measures. Biases in reporting were larger in overweight adolescents than in normal weight or underweight adolescents. Use of a correction formula seems reasonable in order to correct for differences between self-reported and measured data. Body perception might be used to improve the validity of such a formula. The collection of body perception data is therefore recommended in addition to self-reports. If possible, data on the parents' height and weight should also be collected. One possible correction procedure for estimates of the prevalence of overweight and obesity is proposed in Kurth and Ellert (2010) [42]. The development of a correction factor at the individual level based on the existing data is planned for the future. This would improve not only the estimation of the prevalence of over- and underweight in future surveys, but also the assessment of the association between the individual BMI and other variables.

## Abbreviations

BMI: Body mass index; SES: Socio-economic status; WHO: World Health Organisation; CI: Confidence interval

## Competing interests

The authors declare that they have no competing interests.

## Authors' contributions

AKB performed the statistical analysis. ASR and UE were involved in the study-design and data-validation process and counselled and supported AKB on statistical questions. ASR, UE and AKB interpreted the results and AKB wrote the first draft of the manuscript. ASR and UE were involved in writing the manuscript, made substantial contributions and revised the manuscript critically. All authors have read and approved the final version.
